# Influence of Powder Deposition on Powder Bed and Specimen Properties

**DOI:** 10.3390/ma12020297

**Published:** 2019-01-18

**Authors:** Steffen Beitz, Roland Uerlich, Tjorben Bokelmann, Alexander Diener, Thomas Vietor, Arno Kwade

**Affiliations:** 1 Institute for Particle Technology, TU Braunschweig, 38104 Braunschweig, Germany; alexander.diener@tu-braunschweig.de (A.D.); a.kwade@tu-braunschweig.de (A.K.); 2 Institute for Engineering Design, TU Braunschweig, 38106 Braunschweig, Germany; r.uerlich@tu-braunschweig.de (R.U.); t.bokelmann@tu-braunschweig.de (T.B.); t.vietor@tu-braunschweig.de (T.V.)

**Keywords:** additive manufacturing, selective laser sintering, surface roughness, blade geometry, powder application direction, PA12

## Abstract

Three-dimensional printing used to be a rapid prototyping process, but nowadays it is establishing as an additive manufacturing (AM) process. One of these AM techniques is selective laser sintering (SLS), which most often involves partial melting of the particles and therefore belongs to the category of powder bed fusion processes. Much progress has been made in this field by research on process parameters like laser power, hatch distance, and scanning speed while still lacking a fundamental understanding of the powder deposition and its influence on parts. A critical issue for economic manufacturing is the building time of parts with good mechanical properties, which can be reduced by lower surface roughness due to less or missing post processing. Therefore, the influence of three blade shapes on powder bed surface roughness has been evaluated for PA12 powder with three different grain size distributions by using advanced X-ray micro computed tomography (XMT) and a confocal laser scanning microscope (LSM). Along with those methods, new techniques for powder characterization were tested and compared. Lowest roughness has been achieved with a flat blade, based on a higher compression due to a larger contact zone between blade and powder bed. Furthermore, an anisotropic effect of the mechanical properties resulting from different building directions has been detected which can be explained by varying amounts of solid contact paths through the powder bed depending on powder application direction. In addition, an optimal combination of process parameters with an even compression of the powder bed leads to low surface roughness, complementing the advantages of additive manufacturing.

## 1. Introduction

In the last decade, much progress has been made in the field of additive manufacturing (AM), resulting in large-scale industrial applications as well as prototyping with low-cost kits for personal use. A new methodological framework for design guidelines of additive manufacturing (DfAM) is to exploit the potential of this manufacturing process [1]. Among the many AM techniques which are classified in EN ISO/ASTM 52900 [2], this paper focusses on powder bed fusion processes, especially selective laser sintering (SLS), which is described as having the highest potential for breaking the barrier from rapid prototyping (RP) to AM [3]. This method is characterized by partially melting particles at their contacts using a laser to create a solid bond.

Although there has been much investigation on the influence of process parameters and material compounds on the mechanical properties of polymers such as PA12 [4,5,6,7], the effect of the powder deposition process is not yet well understood. For this purpose, a number of experimental and numerical related approaches helped to gain new insights into the topic of creating dense packings and made it possible to better evaluate the powder flow properties [8,9,10,11,12]. Further investigations, such as the determination of appropriate particle sizes for SLS [13] and particle size distributions (PSD) [10], along with new ways of quantifying powder flow properties [12,14] showed that powder characterization still lacks appropriate methods.

A further important feature is the surface quality of the component. When already meeting the product requirements regarding haptics, tribology, or surface roughness after additive production, post-processing such as grinding or polishing is no longer necessary. On the one hand, this reduces the production time and costs, which is essential for the efficiency of AM. On the other hand, private users would benefit from the omission of post-processing.

This paper investigates the effect of powder deposition with three different blade geometries on surface roughness which has not been the focus of recent research, yet. For this purpose, differently sized PA12 powders were applied with a flat, round and sharp blade. A laser scanning microscope (LSM) and an X-ray micro computed tomography (XMT) were used for determination of different arithmetic and average surface roughness of the powder bed. The manufactured specimens were tested with regard to their mechanical properties such as tensile strength, Young’s modulus and fracture strain. Furthermore, XMT coupled with algorithms was used to gather information about the internal structure of the powder beds. Unlike other nondestructive methods like ultrasonic porosity determination [15] the proposed XMT analysis procedure allows the evaluation of the arrangement of particles. 

## 2. Theory

**Figure 1:**
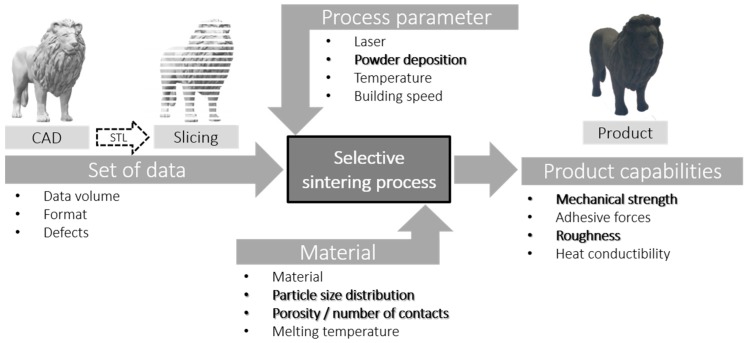
Influencing and in this paper analyzed variables and product properties in selective laser sintering (SLS).

SLS of components results not only in a higher degree of design freedom, but also in process-specific features which have to be considered for optimum process control and thus, component performance. The quality of the components produced is largely determined by the humidity, temperature, gas flow/surrounding atmosphere, material selection, powder state, powder properties, layer thickness, laser parameters, component orientation during printing, and the quality of the data set used (Figure 1) [16,17,18]. Most of those parameters are presented in the following chapters, while this paper endeavors to study the items in bold (Figure 1) in further detail.

### 2.1. Process Parameters

One key parameter for the production of components with homogeneous properties is a uniform temperature distribution within the powder bed. In addition, the sinter window—a temperature range between melting and crystallization point—must also be maintained. The precise adjustment of the temperature in the entire area of the powder bed is associated with considerable difficulties, so that temperature differences of 10 Kelvin can currently be found even in precise systems, especially in the peripheral areas [4,19,20]. The sintering process is followed by a controlled cooling process. Uneven cooling of the component leads to increased internal stresses and thus, to poorer product quality. In addition to temperature, other process parameters also directly influence the quality of the product. For the targeted process control it is necessary to determine the influence of the process parameters and to determine the interaction of process parameters with each other [16].

The most characteristic value in powder bed fusion processes is the so-called energy density ED which includes the parameters laser power LP, scan speed SS, and hatching distance HD [5]. Here the applied energy is related to a surface. By adding layer thickness LT, the ED considers the total energy input per volume [21]:

(1) $$\mathrm{ED}=\;\frac{\mathrm{LP}}{\mathrm{SS}\times \mathrm{HD}\times \mathrm{LT}}$$

Process parameters do not only have an effect on the sintering depth and mechanical properties [22], but also on the surface structure of components close to the final contour. This can be described e.g., by the arithmetic roughness R_a_ and the average surface roughness R_z_. They are defined in DIN EN ISO 4287-07 [23] as:

(2) $$R_{a}=\;\frac{1}{l_{n}}\overset{l_{n}}{\underset{0}{\int }}\left|z\left(x\right)\right|\mathrm{dx}$$

with the length of the measuring section l_n_ and the distance of the profile from the center line z(x) and:

(3) $$R_{z}=\;\frac{1}{m}\;\overset{m}{\underset{i=1}{\sum }}R_{z}\left(i\right)$$

with the individual average surface roughness R_z_(i) in the five sections i (m = 5), which describes the distance between the largest profile maximum and the smallest profile minimum.

Mierzejewska et al. [24] determined by experimental investigations that a reduction of the LT results in smaller roughness values. In the case of larger thicknesses, the “stair step effect” is used. Further empirical studies for the parameters LP (75–150 W), HD (25–100 µm), SS (200–290 mm/s), pulse time (25–100 µs) and powder bed temperature (172–178 °C) were conducted by Sachdeva et al. [25] and Krol et al. [26]. The arithmetic roughness R_a_ as a function of LP and SS mainly shows a concave, parabolic course. The lowest roughness values were achieved at a pulse time of 25 µs, HD 25 µm, laser power 150 W and SS 290 mm/s. A powder bed temperature of 175 °C and long hatch lengths complete the parameter set for low roughness. In general, a high interaction between the process parameters was observed [25,26].

Another approach is the optimization of energy application strategy in terms of time and quality. A method for optimizing the contours was presented by Luo et al. [27] in the form of two algorithms. The first one is called “Scan Pack Control” and adapts the transitions between the scan lines to the requirements of the process at constant speed. Thus, the end points of the lines are no longer approached by the shortest path, but by a speed-dependent trajectory. This procedure avoids high energy densities and improves the quality of the edges. The same principle is used for corner points by a loop, which connects the corner points with a curve instead of a sudden speed and direction change. The second algorithm adjusts the laser parameters pulse frequency, pulse width and power to the variable speed for creating a uniform energy density and thus, homogeneous structures within a layer. These methods can be combined for reducing manufacturing time by 23%.

In addition, Ajoku et al. [28] discussed the “end-of-vector effect” which can be explained by the initial burst of energy every time the laser is activated. Although the LP stabilizes after a few milliseconds this effect is most eminent in small parts where the smallest dimension of the part is placed perpendicular to the scanning direction. Gibson and Shi [29] observed similar results while investigating different build orientations.

**Figure 2:**
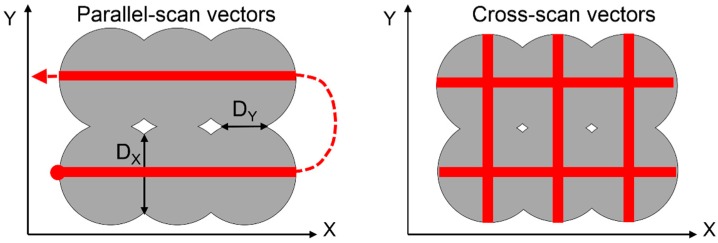
Dimension of necking diameters in relation to the applied scan vectors (modificated 28).

The SS and heat conductivity are also crucial for the dimension of the particle-particle bonds which can be described by the necking diameter D. Figure 2 shows the principle of the necking diameter according to Ajoku et al. [28] where D_X_ is larger than D_Y_ due to cooling processes that takes place between the two parallel-scan vectors. This can cause anisotropic part properties. Starr et al. [30] found out that by increasing the LP the orientation of the parts does not have an impact on these properties even at parallel-scanning strategy. In addition, Kaddar [31] examined different scan strategies regarding their influence on mechanical properties. As a result, cross-scanning with high fill scan counts was found to be the best approach for manufacturing parts with as good as possible isotropic mechanical properties.

Another major build parameter is orientation of the parts in the powder bed [17]. While vertical orientation (Z-direction) of samples result in low tensile strengths, high elongation at break and lower Young’s modulus [17,29,32], the deviation of the results is lower compared to other orientations according to [28]. In contrast, the investigations of Wegner et al. [33] showed that elongation at break is the lowest in vertical build orientation. It was also found out that flat part orientation in the horizontal X-Y-plane does not cause anisotropic effects when cross-scanning in an industrial SLS machine equipped with a roller is used. Regarding parallel-scanning, Sabelle et al. [34] showed that monolayers, made from copper alloy, show the highest tensile strength when the scan vectors are 30° inclined against material testing direction. Investigations on possible build orientations in the Y-Z-plane led to similar results for polymers [35].

Although build orientation and powder deposition go hand in hand, the direction of powder deposition is often not considered in those publications. Although van den Eynde et al. [9] pointed out that the geometry of the spreading blade has an influence, hardly anything can be found regarding blade geometry. An explanation for this lack of knowledge could be the assumption that cross-scanning strategy compensates possible anisotropies in the powder bed [31]. Furthermore, the experiments were performed on industrial machines with rollers as spreading tools. To the best knowledge of the authors low-cost machines and the anisotropic effect of powder deposition with blades were not investigated, yet.

**Figure 3:**
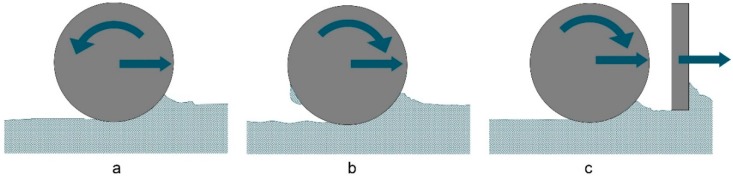
(**a**) Counter-rotating roller; (**b**) forward-rotating roller causing defects in the powder bed; and (**c**) combination of blade and roller (modificated 36).

Budding et al. [36] carried out research on the deposition of gypsum powder regarding the achieved compaction of the powder bed. The use of both, blade and counter-rotating roller, results in good powder bed qualities while the roller is creating a better compaction (Figure 3a). The density could be enhanced by increasing the roller diameter or powder deposition with a forward-rotating roller. However, in the latter case the powder bed was not spread evenly because some of the more cohesive gypsum powder stuck to the roller (Figure 3b). The combination of blade and roller (Figure 3c) was found to be promising for high compaction but needs further investigation.

On the one hand, Drummer et al. [37] showed that an increase of translational deposition speed from 80 mm/s to 120 mm/s leads to a slight increase of the specimen’s density which they explain by higher compression forces. On the other hand, experimental and simulation results of Haeri et al. [8] revealed that decreasing translational velocity increases powder bed quality. In addition, the best outcome was also observed with rollers because of the large contact area and therefore better support between particles and roller. Shanjani and Toyserkani [38] approached the powder spreading and compaction behavior with mathematical modeling concerning e.g., LT and roller diameter.

Instead of the common mechanisms for filling the powder bed with rollers or blades, new techniques were the focus of research as well. Pipettes with different geometries were examined by Kumar et al. [39]. For fine powders (10–25 µm) vibration using piezoelectric strip actuators was advantageous. Furthermore, this method has the advantage that the flow can be stopped without time delay by deactivating the actuator.

### 2.2. Material Characteristics

In the last decade, much progress was made in the field of feed material characterization in the context of AM. For instance, the particle shape influences the flowability, density of the powder bed, as well as the porosity and surface roughness of the component [16]. It can be seen that bulk density of a packing of spherical particles is much higher, compared to those of e.g., flake powders [40]. Haeri et al. [8] identified that higher aspect ratios of the single particles lead to lower surface quality. Furthermore, it was confirmed that spherical particles are easier to handle during the powder layer spreading process [9,12]. Berretta et al. [41] evaluated the particle shape regarding their circularity, aspect ratio, and solidity and discussed their impact on flow behavior.

The state of the powder is another important aspect. It was observed that the use of recycled powder leads to increased porosity and roughness of parts, known as “orange peel” [42]. This effect can be reduced by a higher proportion of new powder or adapted process parameters. Recycling of powder was also investigated by Pham et al. [43] and Dotchev et al. [44]. Furthermore, moisture content can also change the state of the powder. Humidity does not only affect the flow properties [45] but the wettability [46] as well.

It was observed in experiments with different grain sizes, that coarse grains (200 µm) have a better flowability than fine grain powders (63 µm). Due to the higher cohesion between the particles, compared to their gravitational forces, the fine powders were mixed with additives in order to increase their flowability [47]. Furthermore, the normalized packing density was increased to 40.6% compared to 26.6%. Research on the potential of powder compaction was carried out by Greiner et al. [48]. Densification of PA12 leads to higher thermal conductivity and a homogeneous temperature field. A greater layer thickness under the same process parameters was also observed.

Different particle size distributions were also found to have an impact on mechanical properties [49] as well as the flowability of the powder [50]. On the one hand, the removal of fine particles (diameter < 25 µm) enhances the spreadability and therefore the packing density [12]. On the other hand, a certain amount of small particles improves the density of the packing as well as the printed specimens [49]. Based on discrete element method (DEM) simulations a critical particle diameter of 43.6 µm was detected. If the particle diameter is lower, the flowability of the powder decreases [11]. Other studies discussed multimodal PSDs for filling out the voids in order to create a dense packing [51,52]. It has to be pointed out that such values like the critical particle diameter probably differ for varying materials. For instance, gravitational forces on particles will be much higher for a metal powder compared to a polymer powder of the same size.

Yet another important aspect is the quantitative evaluation of powder spreadability or flowability which means the ability to form a smooth powder bed during application. Therefore, Prescott and Barnum [53] suggest distinguishing between the flowability in a specific machine/apparatus and flow properties that rely solely on interactions between particles and are independent of the used devices. A simple method for evaluating powder flow properties is the Hausner ratio (HR) in which the density of the poured powder is put in relation to the tapped powder [54]. Although the HR method was criticized for its lack of applicability to the forced spreading mechanism [9], it is a commonly used technique in AM due to its easy accessibility and good reproducibility [55]. Further popular approaches are the avalanching angle of a powder inside a rotating drum [14,41] and the usage of a powder rheometer. Different characteristic values can be derived from the measured torque [12,56]. Regarding the flowability, van den Eynde et al. [9] developed a material spreading device in order to mimic the powder deposition process. Although shear tests were mentioned for the distinction of flow properties [57] they were not applied systematically to AM processes, because they are evaluated as “not well suited” for AM, as powders are tested in confined state [58]. 

Current research is also focused on finding new suitable polymer materials or blends for AM. Hybrid polymer materials like PA12 and polybutylene terephthalate (PBT) or PA12 and high-density polyethylene (HDPE) were optimized due to higher mechanical performance [6,7]. 

## 3. Materials and Methods

### 3.1. Powders

**Figure 4:**
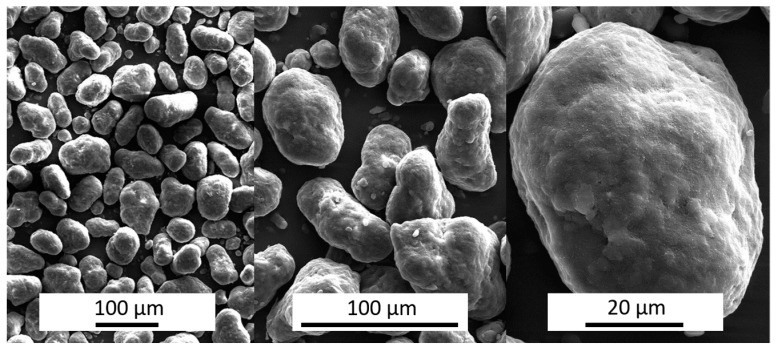
Scanning electron microscope (SEM) images of PA 12 Original powder at 200-, 500-, and 2000-fold magnification.

This study is based on PA 12 powder which is widely used as polymer in selective laser sintering applications [17]. Images of the particles were obtained by a scanning electron microscope (SEM) Helios G4 CX (FEI Company, Hillsboro, OR, USA) at 200-, 500- and 2000-fold magnification (Figure 4). They can be described as having a fairly spherical potato-like shape without any sharp edges.

For the investigation of different powder grades the original powder (PA12 Original) was air sieved with a mesh size of 50 µm (Hosokawa Alpine AG, Augsburg, Germany). The separation resulted in two fractions: PA 12 Coarse and PA 12 Fine.

PSD of the powder fractions were determined by both laser diffraction and dynamic image analysis. The aforementioned measurements via laser diffraction were carried out using a HELOS particle size analyzer with a dry dispersing unit (Sympatec GmbH, Clausthal-Zellerfeld, Germany). This method is based on the analysis of typical diffraction patterns resulting from the interaction of the laser light with the particles. As suggested in the guideline VDI 3405 1.1 [59] the whole PSD was determined with special respect to the x_10_, x_50_ and x_90_ particle diameters (Table 1). 

**Figure 5:**
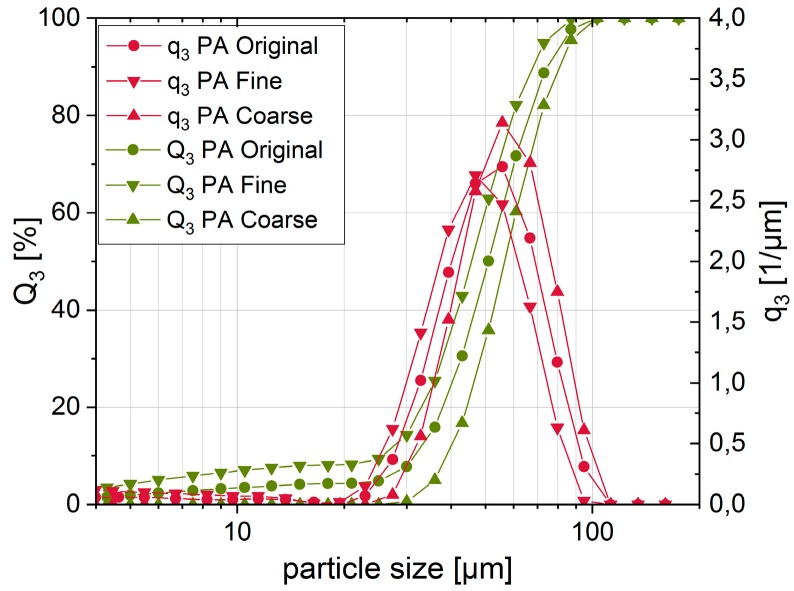
Cumulative fraction Q_3_ and density distribution q_3_ of particle size distributions (PSD) of PA by dynamic laser diffraction.

Figure 5 shows the cumulative fraction Q_3_ and the density distribution q_3_ of the particle sizes. It can be seen that the slope of the different fractions is very similar and mostly only the particle sizes are shifted towards smaller or bigger sizes. The coarse fraction shows the narrowest PSD. In contrast, the original and the fine PA fractions include a detectable amount of fine particles below 20 µm.

As an alternative method, particles were also analyzed by a dynamic image analysis system (QICPIC, Sympatec GmbH, Clausthal-Zellerfeld, Germany). Therefore, particles are dispersed by a vibrating feeder and move gravimetrically through the image plane. Along the way, several images of the particles are taken by a high speed camera under pulsed light conditions. As a result, not only the size but also the projected 2-D shape of the single particles can be displayed. From this data the sphericity ψ is derived by putting the calculated perimeter P_EQPC_ of a circle with the same area as the projected area of the particle in relation to its real perimeter P_R_:

(4) $$\psi =\frac{P_{\mathrm{EQPC}}}{P_{R}}$$

The closer the sphericity is to one the more spherical the particles are shaped. The particles have a median sphericity of more than 0.85 which confirms the impression from the randomly monitored SEM images.

Furthermore, different methods of classical powder characterization were applied for distinguishing the powder properties. In a first step, bulk density ρ_b_ and tap density ρ_t_ were experimentally determined according to DIN ISO 697 [60] and a tapping device according to DIN EN ISO 3953, respectively [61]. Both powder density experiments were repeated five times.

Regarding their flow properties the powders were examined by using HR as a quotient of tap and bulk density (Table 2). If the dimensionless value is between 1 and 1.25, the powder is considered well flowing [56]. It can be seen that the original powder shows slightly higher density values because smaller particles fill out the voids between bigger particles. All fractions can be classified as well flowing by using the HR method. In general, there are only minor differences in the HR values which can be considered as equal regarding the fact that the tapping method is not a very sensitive method.

### 3.2. SLS System

**Figure 6:**
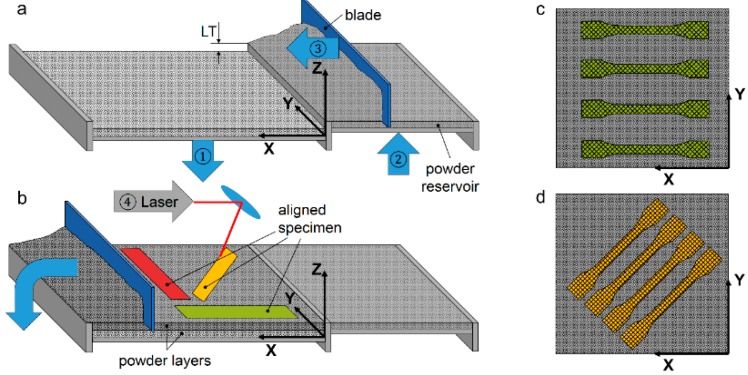
(**a**) Adjusting layer thickness LT and powder deposition; (**b**) removal of excessive material and sintering of specimens; (**c**) and (**d**) schematic arrangement of x- and x-y-specimens and scan vectors.

SLS was carried out by the Sintratec Kit with the parameters shown in Table 3 which result in an ED of 0.116 kJ/mm^3^. In preparation of the sintering process the powder is heated to 171 °C first. Then the blade moves along the surface of the powder bed as seen in the Figure 6a. Afterwards the excessive powder is removed and the laser scans the powder bed surface. The specimens were built by varying orientations in the X-Y-plane (Figure 6b). Cross- and outline-scan strategy were used for energy application routes (Figure 6c,d). All tensile specimens were manufactured using a flat blade and PA Original. 

**Figure 7:**
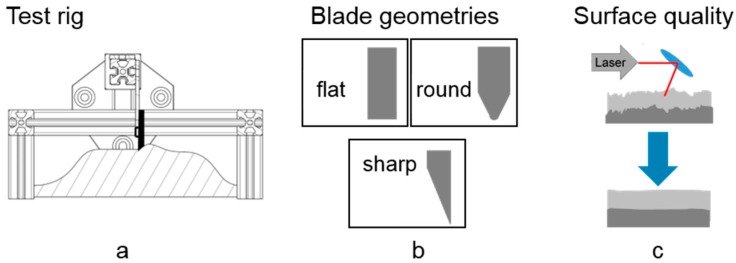
(**a**) Test rig; (**b**) different blade shapes; (**c**) possible powder bed and specimen surface profiles.

Moreover, the influence of the cross-sectional shape of the blades has been investigated using the Sintratec Kit as well as a custom spreading test rig (Figure 7a). Therefore, three different geometries were utilized (Figure 7b), while the original blade has a flat bottom and the other ones were modified regarding their edge geometry.

### 3.3. Powder Bed and Mechanical Tests

**Figure 8:**
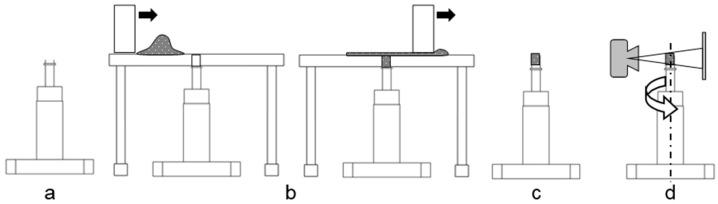
(**a**) Insert with mount for X-ray micro computed tomography (XMT); (**b**) insert with powder deposition plate and powder application; (**c**) insert with cover; (**d**) tomography process.

For the investigation of the surface quality of the powder bed, two test rigs have been designed: a lab-scale experimental setup (Figure 7a) and an insert (Figure 8a) which can be put inside the SLS system and an XMT for further analysis. Powders were applied under room conditions for better comparability and because the SLS chamber temperature cannot be maintained inside the XMT or LSM. Furthermore, this course of action prevents settling of the particles due to shrinking during the long time image acquisition process. The XMT and LSM could not be integrated inside the SLS chamber, either.

The powder spread by the lab-scale setup is analyzed contactless by an LSM (KEYENCE VK-9710, Keyence Corporation, Osaka, Japan) where the light beam illuminates the surface and is diffracted so that it hits the lens of the objective and is focused in the intermediate image. Since not all beams have their focus in this plane, the image around the focused point is blurred. This blur is filtered through a pinhole in the intermediate plane to reveal only the focus point. Therefore, the light intensity in the objective decreases, which can be considerably increased by using a laser source. With the aid of galvano mirrors, the specimens are scanned two- and even three-dimensionally through the axially movable objective lens. The light beams are then filtered through the pinhole and converted into electrical signals by the sensors [62].

For this test case, an area of 5 mm × 5 mm was determined centrally and the topology of the powder surface was recorded. The three-dimensional surface profile was filtered with regard to interference effects such as noise and inclination. Afterwards, the line roughness in both the powder application direction of the blade and perpendicular to it was determined. They have been analyzed in accordance with DIN EN ISO 4287 [23]. The arithmetic roughness R_a_ and the average surface roughness R_z_ were determined at five lines each in X- and Y-direction for the three blade geometries (flat, round and sharp) and the three powder grades (fine, original and coarse) resulting in nine combinations of them.

The powder bed spread by the SLS system was analyzed by using XMT and post-processing of the acquired images. X-ray source and detector were kept close to the sample in order to ensure a sufficient resolution for proper single particle segmentation in the post-processing step. This results in a sample size of about 1.8 mm. For powder spreading, a custom sample holder insert (Figure 8a) in combination with a powder deposition plate (Figure 8b) was put on the building platform of SLS system. The edges of the sample pot were adjusted to be flush with the powder deposition plate. In the next step, one thin layer of powder was spread across the plate filling the sample holder.

Afterwards, plate and insert were carefully removed to prevent vibration and thus, undesired particle rearrangement. For further protection, a cover (Figure 8c) was put cautiously on top of the insert and the mount was placed inside the XMT (Xradia XCT 400, Carl Zeiss AG, Oberkochen, Germany). Then images around 184° of the powder sample’s vertical axis were taken (Figure 8d). Additional information about the XMT configuration is shown in Table 4.

**Figure 9:**
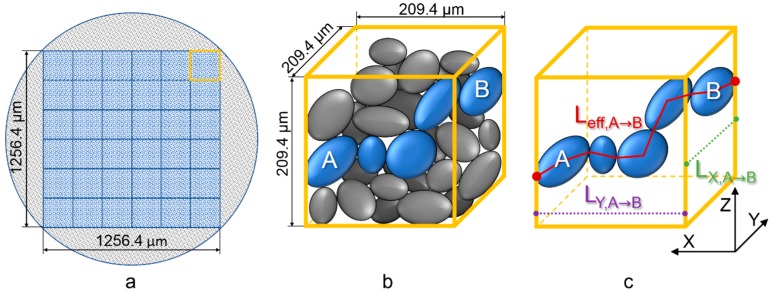
(**a**) Cropping of horizontal slices to squares and dividing the volume into cubes; (**b**) view at showcase cube filled with particles and illustration of the shortest solid path of two particles (A and B); (**c**) illustration of efficient (L_eff_) and ideal solid path lengths (L_X/Y_) via skeletal contact modeling between the particles in different axes.

The raw data were post-processed after measuring. For maximum contrast, beam hardening and center shift correction were applied to cross-section images. The horizontal images were each filtered (Avizo Fire 7, FEI Company, Hillsboro, OR, USA) by non-local means and cropped to a square format with an edge length of 1256.4 µm (Figure 9a).

Subsequently, the particles were segmented from the surrounding air by a Matlab code based on the algorithm of Otsu [63] and little objects were removed. Then, the volume was divided into 36 cubes with an edge length of 209.4 µm each (Figure 9b).

Afterwards, a skeleton model was derived according to Lee et al. [64]. One skeletal route for the path from center of particle A to B is illustrated in Figure 9c in which the ideal distance along the X- or Y-axis between the particles is described as L_X/Y,A__→B_ whereas L_eff,A__→B_ is the effective length of the solid path. Subsequently, the solid tortuosity can be calculated with the following equation:

(5) $$T_{S}=\frac{L_{\mathrm{eff},A\to B}}{L_{X/Y,A\to B}}$$

Originally, tortuosity was implemented for modeling properties of porous media due to different pore properties [65]. In this paper the focus is on mechanical properties of powder beds and sintered parts. Therefore, a solid tortuosity T_S_ was derived from the particle contacts (Figure 9c). In addition, more data can be derived from the XMT measurements like porosity distributions and the powder bed surface information.

For determination of the mechanical properties of the sintered specimens universal testing machine (Zwick Roell, ZwickRoell GmbH & Co. KG, Ulm, Germany) was used. According to DIN EN ISO 527-1 the Young’s moduli were derived from the force-displacement curves [66]. The tensile strength and fracture strain under stress were likewise obtained.

## 4. Results

### 4.1. Powder Bed Characterization

**Figure 10:**
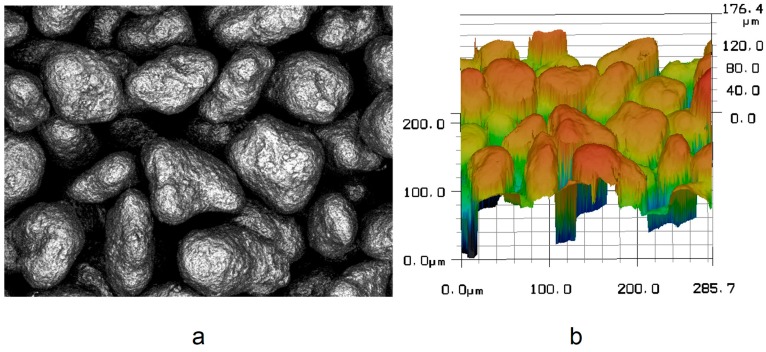
(**a**) Excerpt of an LSM scanning process and (**b**) Excerpt of the surface topology data set.

The roughnesses R_z_ and R_a_ of the powder bed have been measured using both LSM and XMT. Each data point of an LSM scan (Figure 10a) includes a height value in Z-direction. This data grid (Figure 10b) represents the surface topology and only gives information about the highest peaks at every X-Y-position.

**Figure 11:**
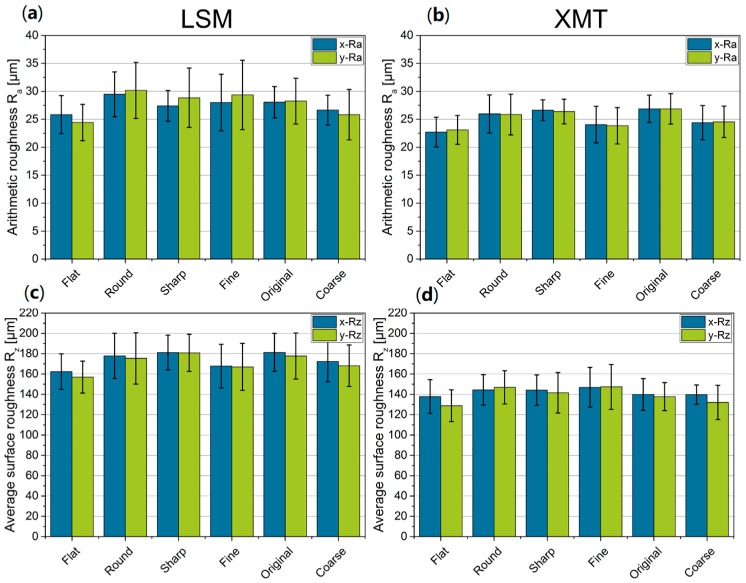
Mean arithmetic and average surface roughness of powders in deposition and orthogonal direction depending on blade geometry determined by LSM (**a**,**c**) and micro XMT (**b**,**d**).

It has been observed that the lowest arithmetic roughness (LSM: approx. 25 µm, XMT: approx. 23 µm) was achieved with a flat bottom geometry of the blade (Figure 11a,b). This results from the higher compression of the powder bed, which is greater due to the two-dimensional contact zone. A round or sharp blade reduces the effective vertical compression force, so that the surface roughness increases to 26 µm or 27 µm, measured by LSM.

The influence of powder composition and blade geometry on average surface roughness measured by LSM is identical to that of the arithmetic roughness (Figure 11c,d). At approx. 170 µm, powder beds of fine and coarse particles have lower values than the original powder at 180 µm. With regard to the flat blade, a low roughness of the powder bed of approx. 162 µm was achieved compared to the round and sharp blade shape of approx. 180 µm. A significant difference between the application direction of the powder and perpendicular to it could not be observed.

**Figure A1:**
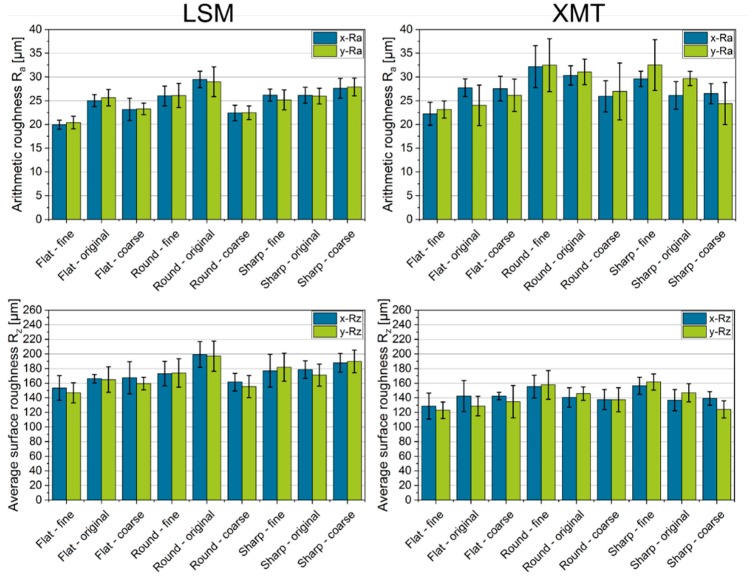
Roughness values for each tested combination of blade and powder fraction.

Compared to the measuring method with the LSM, the roughness values obtained from XMT data are considerably different (R_a_ ≈ +3 µm and R_z_ ≈ −20 µm) (Figure 11a–d), which can be attributed not only to the method but also to the smaller sample size which results in lower values along the section of measurements for the average surface roughness. A smaller measuring length leads to higher values for the arithmetic roughness because peaks cannot be leveled out. This can also be seen in the higher deviations of R_a_ from XMT data. The authors assume that enlarging the analyzed area and therefore, the sample in the XMT would decrease the deviations. In addition, Figure A1 in the Appendix shows the results for the roughness of each combination of blade and grade. Although the powders were applied and measured differently, both methods agree with each other which can be seen for instance in the low roughness of the coarse powder applied with the round blade compared to the other grades.

**Figure A2:**
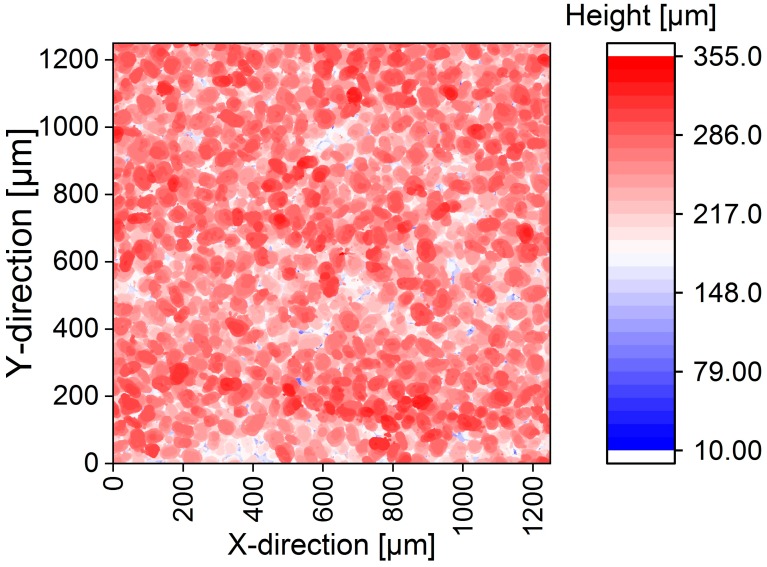
Topology of a powder bed (flat blade, PA Original) measured with XMT.

However, it can also be seen that there is no significant general difference in roughness between the fine and coarse powder. An effect of the powder deposition direction could not be detected, either. This was confirmed by both optical impression and the data set obtained from the measurements (Figure A2) which have shown no differences in roughness values. Furthermore, it has to be pointed out that the powder bed has been prepared by the test rig for the LSM measurements. Even if the powder deposition process is the same, we cannot exclude slight differences in the systems like translational movement. In addition, all powders have not been spread at elevated powder bed temperatures (>140 °C) neither in the test rig nor in the SLS system because those temperatures cannot be preserved during XMT and LSM measurements.

Differences in the solid path were observed regarding the internal structure of the original powder applied with the flat blade. This combination was also applied for the manufactured specimens). Although the average solid tortuosity in powder deposition direction (T_x_ = 2.34 ± 0.37) does not differ from the solid tortuosity perpendicular to it (T_y_ = 2.36 ± 0.34), it can be seen that the amount of detected solids paths through the powder bed is 16% higher in the direction of powder deposition. More than 300 solids paths were found in powder deposition direction while slightly more than 260 paths were determined in Y-direction powder application direction affects the arrangement of particles to each other. 

**Figure 12:**
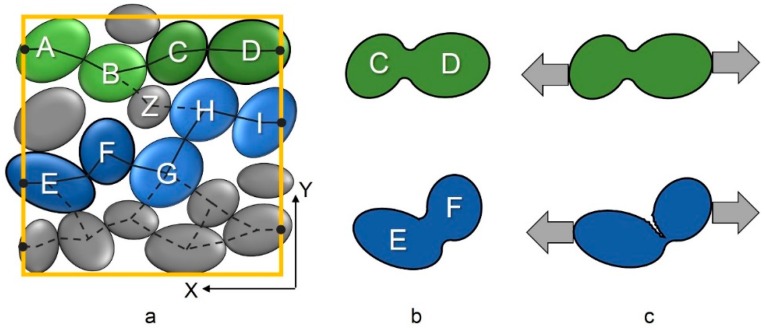
(**a**) Possible solid paths in X-direction in one analyzed square; (**b**) sintered particles of paths A→D and E→I; and (**c**) possible reactions of sintered particles when stressed in X-direction.

Another characteristic value is the average of the best solid tortuosities of all analyzed cubes. This is visualized schematically for a square in Figure 12a. In this example, solid path A→D has the lowest and therefore best T_S_ in X-direction whereas T_S_ for path E→I is higher. Assuming that particles along those solid paths are bonded together (Figure 12b) it can be concluded that their reaction to stress in different directions might vary (Figure 12c). Long single paths consist of more particles and therefore more weak points between them. All in all, solid path A→D should exhibit superior strength compared to the other paths. But in practice, there are cross-links between different particles and solid paths. For instance, particle Z connects both aforementioned solid paths with each other. This way, path A→D strengthens the whole structure inside the examined volume (Figure 12a).

For the experiments, it was observed that powder deposition direction is slightly more favorable with T_S,X,best,mean_ = 2 compared to T_S,Y,best,mean_ = 2.18. In regard to cross-linking, such a little difference in the mean best solid tortuosity probably has only little impact on mechanical strength of specimens. As a result, the aforementioned amount of solid paths seems to be more important because of more cross-linked contacts. 

### 4.2. Specimen Characterization

**Figure 13:**
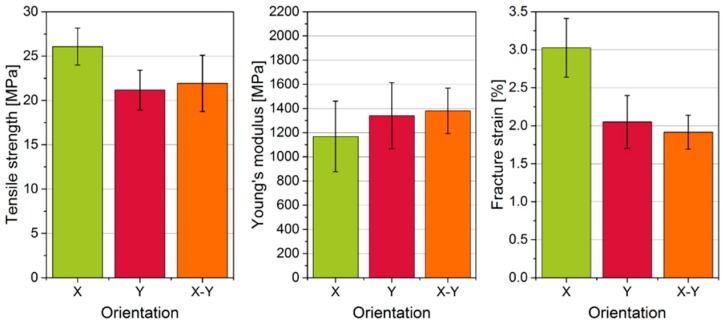
Tensile strength, Young’s modulus and fracture strain of differently aligned specimens in the SLS machine.

The results of the mechanical properties of the manufactured specimens are shown in Figure 13. Both the tensile strength (X: 26.1 N/mm, Y and XY < 22 N/mm²) and the fracture strain (X: 3%, Y and XY < 2.1%) are considerably higher for specimens that are aligned in powder deposition direction (X). Only the Young´s modulus of the parts in Y- or X-Y-direction are slightly higher than in X-direction, but the differences are within the standard deviations. Based on previous research, it could be expected that no such anisotropic behavior should be observed because cross-scan strategy was utilized [31]. When compared to mechanical properties of commercially processed PA 12 powder [67], it has to be pointed out that the achieved tensile strengths of specimens even when manufactured in powder deposition direction are lower than of those manufactured on industrial SLS systems. The same applies for the fracture strain and the Young’s modulus. A reason for this is that low-cost SLS systems cannot provide advanced parameter controls like an even temperature distribution inside the building chamber which in turn affects e.g., reproducibility. Previous research of Spoerk et al. [68] reported that an altering of chamber temperature leads to decrease the elongation at break and the impact energy. Additionally, they found that altering of the chamber temperature results in changes within the particle morphology concerning the spherulite size and the crystal modification. Both impacts enhance the dimensional accuracy in the case of warpage and surface quality.

The anisotropy of mechanical properties can be explained by the higher amount of particle contacts along the powder deposition direction: 16% more solids paths in X- than in Y-direction result in 18.6% higher tensile strengths. The possibility of particles being bound together by sintering is higher when those particles are already in contact with each other. Orientation in the intermediate X-Y-direction in the horizontal plane does not show major difference compared to Y-direction. This means that the correspondence of build and powder deposition direction has the biggest influence on mechanical properties. However, we cannot fully exclude that the laser source of the low-cost SLS system also has an influence on this anisotropy. According to research carried out on industrial machines equipped with rollers, different build orientations in the horizontal plane do not have such a great impact on their mechanical properties when using cross scanning method [31]. Another reason for the higher tensile strength could be the possible alignment of non-spherical particles similar to the observation Haeri et al. [8]. made. This could lead to fewer contacts and therefore less predetermined breaking points in direction of powder deposition. All in all, specimens made from a low-cost SLS system do not only have inferior quality compared to industrial machines but also need to be placed in the right build orientation in correspondence with powder deposition for best mechanical properties. 

## 5. Conclusions

In this paper, the influence of material and process parameters on powder bed and product properties was investigated by processing three fractions of PA 12 on a low-cost SLS system and a test rig. It was shown that the surface quality of a powder bed is influenced by the shape of the blade which was used for powder application. A flat bottom shape was found to be more favorable compared to sharp and slightly rounded edges. The greater horizontal contact zone between blade and powder bed leads to an even compression which results in a more uniform and dense powder bed. In contradiction, the three fractions of PA 12 did not differ strongly in the resulting surface quality. This was confirmed by roughness measurements in powder deposition direction and perpendicular to it. For that purpose an LSM and an XMT were utilized and compared.

This study also revealed that direction of powder deposition can affect mechanical properties of manufactured parts but not the surface quality of the powder bed. Horizontally aligned tensile specimen that were tested in direction of powder deposition have exhibited more than 18% higher tensile strength and almost 50% greater fracture strain while Young’s modulus decreased compared to other combinations of build and deposition direction.

For further investigation an adapted algorithm was introduced for the detection of solid paths through the powder bed which are described by the connection of particle contacts in the powder bed. The amount of solid paths was shown to be higher in the direction of powder deposition which agrees with the aforementioned findings on the mechanical properties.

## 6. Outlook

The lack of suitable materials for AM urges research. Enhanced properties could be achieved by surface modification of powders resulting in e.g., higher laser light absorptivity or better flow properties und thus, spreadability. Furthermore, different blends of powders with different or multimodal PSDs for creating more dense packings as well as more solid paths throughout the powder bed could be investigated with the proposed methods in order to develop particle based models for predicting the packing of powder beds as a function of powder (e.g., inter particle friction) and deposition parameters (e.g., spreading speed). Further structural analysis with advanced algorithms along with numerical approaches could help to improve understanding the impact of induced anisotropic arrangements on powder bed and product properties.

For this purpose, the basic powder application process and actual applied stresses needs further investigation. Further geometries, their limits and their influence on powder bed compaction have to be explored. Along with that, appropriate flowabilty characterization techniques can be developed in order to predict the spreadability in a powder bed fusion system. Shear testing might be a suitable method since there are already established devices which provide a good reproducibility. When the real shear stress between blade and powder bed is known, adapted configurations and procedures might lead to better results for comparing different powders.

Furthermore, low-cost SLS systems are becoming more and more popular for personal use. Besides research on industrial machines the focus needs also to be put on feasible improvements in this sector in order to improve these processes for private purposes and thus, making them more accessible. 

## Figures and Tables

**Table 1:** Specific particle sizes of the fractions.

Size	PA Original	PA Fine	PA Coarse
x_10_ (µm)	32.08	26.11	38.98
x_50_ (µm)	51.14	46.03	56.80
x_90_ (µm)	74.99	68.58	81.19

**Table 2:** Bulk density, tap density and Hausner ratio of the PA fractions.

Powder Characteristics	PA Original	PA Fine	PA Coarse
ρ_b_ (kg/m^3^)	487.7	478.6	482.9
ρ_t_ (kg/m^3^)	569.3	567.8	563.0
Hausner ratio (-)	1.167	1.186	1.166

**Table 3:** Process parameters during SLS.

Process Parameter	Value
Brand	Sintratec AG, Switzerland, Brugg
Type	Sintratec Kit
Temperature-powder bed	171 °C
Temperature-chamber	147 °C
Laser source	Diode Laser–2.3 W–445 nm
Laser Speed	550 mm/s
Focus diameter	0.25 mm
Layer thickness	120 µm
Hatch distance	0.3 mm

**Table 4:** XMT set-up.

XMT Parameter	Value
Number of images	2000
Exposure time	15 s
Magnification	10 x
Voxel size	1.047 µm/voxel
X-ray current intensity	20 µA
X-ray source voltage	50 kV
